# Prostate cancer therapy personalization via multi-modal deep learning on randomized phase III clinical trials

**DOI:** 10.1038/s41746-022-00613-w

**Published:** 2022-06-08

**Authors:** Andre Esteva, Jean Feng, Douwe van der Wal, Shih-Cheng Huang, Jeffry P. Simko, Sandy DeVries, Emmalyn Chen, Edward M. Schaeffer, Todd M. Morgan, Yilun Sun, Amirata Ghorbani, Nikhil Naik, Dhruv Nathawani, Richard Socher, Jeff M. Michalski, Mack Roach, Thomas M. Pisansky, Jedidiah M. Monson, Farah Naz, James Wallace, Michelle J. Ferguson, Jean-Paul Bahary, James Zou, Matthew Lungren, Serena Yeung, Ashley E. Ross, NRG Prostate Cancer AI Consortium, Michael Kucharczyk, Luis Souhami, Leslie Ballas, Christopher A. Peters, Sandy Liu, Alexander G. Balogh, Pamela D. Randolph-Jackson, David L. Schwartz, Michael R. Girvigian, Naoyuki G. Saito, Adam Raben, Rachel A. Rabinovitch, Khalil Katato, Howard M. Sandler, Phuoc T. Tran, Daniel E. Spratt, Stephanie Pugh, Felix Y. Feng, Osama Mohamad

**Affiliations:** 1 Artera, Inc, Mountain View, CA USA; 2 Department of Epidemiology and Biostatistics, University of California San Francisco, San Francisco, CA USA; 3 Department of Biomedical Data Science, Stanford University, Stanford, CA USA; 4 Department of Radiation Oncology, University of California San Francisco, San Francisco, CA USA; 5 NRG Oncology Biospecimen Bank, San Francisco, CA USA; 6 Department of Urology, Northwestern University, Evanston, IL USA; 7 Division of Urologic Oncology, University of Michigan Comprehensive Cancer Center, Ann Arbor, MI USA; 8 Department of Population and Quantitative Health Sciences, Case Western Reserve University, Cleveland, OH USA; 9 Salesforce Research, San Francisco, CA USA; 10 Department of Radiation Oncology, Washington University School of Medicine, Saint Louis, MO USA; 11 Department of Radiation Oncology, Mayo Clinic, Rochester, MN USA; 12 Department of Radiation Oncology, cCare, Fresno, CA USA; 13 Department of Radiation Oncology, Horizon Health Network-Saint John Regional Hospital, Saint John, JB E2L 4L2 CA Canada; 14 Department of Hematology and Oncology, Ingalls Memorial Hospital, Harvey, IL USA; 15 Department of Radiation Oncology, Allan Blair Cancer Centre, Regina, SK S4T 7T1 CA Canada; 16 Department of Radiation Oncology, CHUM - Centre Hospitalier de l’Universite de Montreal, Montreal, QC H2X 3E4 CA Canada; 17 Department of Radiation Oncology, Cedars-Sinai Medical Center, Los Angeles, CA USA; 18 Department of Radiation Oncology, University of Maryland, Baltimore, MD USA; 19 Department of Radiation Oncology, University Hospitals Seidman Cancer Center, Case Western Reserve University, Cleveland, OH USA; 20 NRG Oncology Statistics and Data Management Center, Philadelphia, PA USA; 21 Department of Radiation Oncology, Accrual-Nova Scotia Cancer Centre, Halifax, Nova Scotia B3H 1V7 Canada; 22 Department of Oncology, The Research Institute of the McGill University Health Centre (MUHC), Montreal, QC H4A 3J1 CA Canada; 23 Department of Radiation Oncology, University of Southern California, Los Angeles, CA USA; 24 Department of Radiation Oncology, Northeast Radiation Oncology Centers (NROC), Dunmore, PA USA; 25 Department of Medical Oncology, UCLA Medical Center, Los Angeles, CA USA; 26 Department of Radiation Oncology, Tom Baker Cancer Centre, Calgary, AB T2N 4N2 CA Canada; 27 Department of Radiation Oncology, Washington Cancer Institute, Washington, DC USA; 28 Department of Radiation Oncology, Veteran Affairs New York Harbor Healthcare System-Brooklyn Campus, Brooklyn, NY USA; 29 Department of Radiation Oncology, Kaiser Permanente Los Angeles Medical Center, Los Angeles, CA USA; 30 Department of Radiation Oncology, Indiana University School of Medicine, Indianapolis, IN USA; 31 Department of Radiation Oncology, Christiana Care Health System-Christiana Hospital, Newark, DE USA; 32 Department of Radiation Oncology, University of Colorado Hospital, Aurora, CO USA; 33 Department of Medical Oncology, Hurley Medical Center, Flint, MI USA

**Keywords:** Prognostic markers, Prostate cancer, Machine learning

## Abstract

Prostate cancer is the most frequent cancer in men and a leading cause of cancer death. Determining a patient’s optimal therapy is a challenge, where oncologists must select a therapy with the highest likelihood of success and the lowest likelihood of toxicity. International standards for prognostication rely on non-specific and semi-quantitative tools, commonly leading to over- and under-treatment. Tissue-based molecular biomarkers have attempted to address this, but most have limited validation in prospective randomized trials and expensive processing costs, posing substantial barriers to widespread adoption. There remains a significant need for accurate and scalable tools to support therapy personalization. Here we demonstrate prostate cancer therapy personalization by predicting long-term, clinically relevant outcomes using a multimodal deep learning architecture and train models using clinical data and digital histopathology from prostate biopsies. We train and validate models using five phase III randomized trials conducted across hundreds of clinical centers. Histopathological data was available for 5654 of 7764 randomized patients (71%) with a median follow-up of 11.4 years. Compared to the most common risk-stratification tool—risk groups developed by the National Cancer Center Network (NCCN)—our models have superior discriminatory performance across all endpoints, ranging from 9.2% to 14.6% relative improvement in a held-out validation set. This artificial intelligence-based tool improves prognostication over standard tools and allows oncologists to computationally predict the likeliest outcomes of specific patients to determine optimal treatment. Outfitted with digital scanners and internet access, any clinic could offer such capabilities, enabling global access to therapy personalization.

## Introduction

In 2020, 1,414,259 new cases and 375,304 deaths from prostate cancer occurred worldwide^1^. While prostate cancer is often indolent and treatment can be curative, prostate cancer represents the leading global cause of cancer-associated disability due to the negative effects of over- and under-treatment, and is a leading cause of cancer death in men^2,3^. Determining the optimal course of therapy for an individual patient is difficult, and involves considering their overall health, the characteristics of their cancer, the side effect profiles of many possible treatments, outcomes data from clinical trials involving patient groups with similar diagnoses, and the prognostication of their expected future outcomes. This challenge is compounded by the lack of readily accessible prognostic tools to better risk-stratify patients.

One of the most common systems used to risk-stratify patients worldwide is the National Comprehensive Cancer Network (NCCN), or D’Amico, risk groups developed in the late 1990s. This system is based on digital rectal examination of the prostate, serum prostate-specific antigen (PSA) level, and tumor biopsy grade assessed by histopathology. This three-tier system forms the basis of treatment recommendations used for localized prostate cancer throughout the world^4^, but has repeatedly been shown to have suboptimal prognostic and discriminatory performance^5^. This in part is due to the subjective and non-specific nature of the core variables in these models. For instance, Gleason grading^6^ was developed in the 1960s and has suboptimal interobserver reproducibility even amongst expert urologic pathologists^7,8^. Although newer clinicopathologic risk-stratification systems have been created, three variables remain at their core—Gleason score, T-stage, and PSA^9^.

More recently, tissue-based genomic biomarkers^10^ have demonstrated superior prognostic performance. However, nearly all of these tests lack validation in prospective randomized clinical trials in the intended use population, and there has been little to no adoption outside of the United States due to costs, laboratory requirements, and processing time^11^. Importantly, prognostic models developed on cohort study data and not on randomized clinical trials are subject to selection biases from treatment decisions made in the clinic, and often have less accurate clinical and long-term outcome data. As such, there remains a serious unmet clinical need for improved and more accessible tools to personalize therapy for prostate cancer^12^.

Artificial intelligence (AI) has demonstrated remarkable capabilities across a number of use-cases in medicine, ranging from physician-level diagnostics^13^ to workflow optimization^14^, and has the potential to support cancer therapy.^15,16^ As clinical adoption of digital histopathology continues^17^, AI can be implemented more broadly in the care of cancer patients. Advances and progress in the use of AI for histopathology-based prognostics have already begun, for instance by predicting short-term patient outcomes^18^ or by improving the accuracy of Gleason-based cancer grading on postoperative surgical samples^19^. Whereas standard risk-stratification tools are fixed and based on few variables, AI can learn from large amounts of minimally processed data across various modalities. In contrast to genomic biomarkers, AI systems leveraging digitized images are lower-cost and massively scalable. In addition, these tools can incrementally improve over time through continued learning to optimize test performance and health care value.

In this study, we demonstrate that a multimodal AI (MMAI) system can be used to address an unmet need for accessible and scalable prognostication in localized prostate cancer. This MMAI system has the potential to be a generalizable digital AI biomarker for global adoption. Herein, we train and validate prognostic biomarkers in localized prostate cancer using five NRG Oncology phase III randomized clinical trials by leveraging multimodal deep learning on digital histopathology and clinical data^20–24^. By utilizing data from large clinical trials with long-term follow-up and treatment information that is standardized and less subject to bias, our model learns from and is trained on some of the most accurate clinical and outcome data available.

## Results

We created a unique MMAI architecture that ingests both tabular clinical and image data, and trains with self-supervised learning to leverage the substantial amount of data available. We trained and validated six distinct models on a dataset of 16,204 histopathology slides (~16 TB of image data) and clinical data from 5,654 patients to predict six binary outcomes varying by endpoints and timeframes (5- and 10-year distant metastasis, 5- and 10-year biochemical failure, 10-year prostate cancer-specific survival, and 10-year overall survival). Notably, accurate prediction of distant metastasis at 5 and 10 years is particularly important for identifying patients who may have more aggressive disease and require additional treatment. We measured the performance of these models with the area under the time-dependent receiver operator characteristic curve (AUC) of sensitivity and specificity, based on censored events accounting for competing risks, and the NCCN risk groups served as our baseline comparator. Prior to model development, data from all five clinical trials were split into training (80%) and validation (20%). The MMAI model consistently outperformed the NCCN risk groups across all tested outcomes when comparing the performance results for the validation set.

### Developing the MMAI architecture

**Fig. 1:**
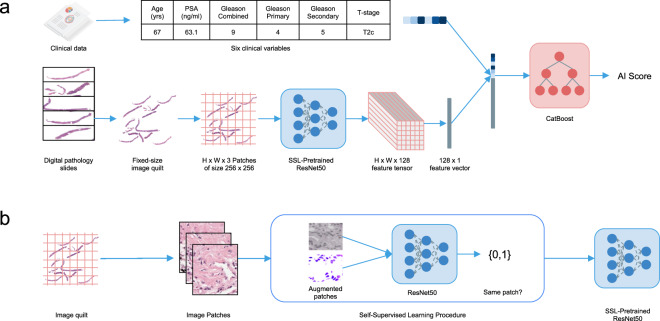
Multimodal deep learning system and dataset. **a** The multimodal architecture is composed of two parts: a tower stack to parse a variable number of digital histopathology slides and another tower stack to merge the resultant features and predict binary outcomes. **b** The training of the self-supervised model of the image tower stack.

For each patient, the MMAI model took as input clinical variables—including the NCCN variables (combined Gleason score, clinical T-stage, baseline PSA), as well as age, Gleason primary, and Gleason secondary—and digitized histopathology slides (median of 2 slides). Joint learning across both data streams is complex and involves building two separate machine learning pipelines—one for learning feature embeddings from the pathologic image data (Image pipeline) and the other to jointly learn from both clinical and image data to output risk scores for an outcome of interest (Fusion pipeline, see Fig. 1a). We standardized the image features across the trials for consistency.

Effective learning of relevant features from a variable number of digitized histopathology slides involves both image standardization and self-supervised pre-training. For each patient, we segmented out all the tissue sections in their biopsy slides, and combined them into a single large image, called an image quilt, of a fixed width and height across all patients (Supplementary Fig. 1). We then overlaid a grid over the image quilt which cut it into patches of size 256 × 256 pixels across its RGB channels. These patches were then used to train a self-supervised learning model^25^ to learn histomorphological features useful for downstream AI tasks (Fig. 1b). Once trained, the self-supervised learning model took the patches of an image quilt and output a 128-dimensional vector representation for each patch. Concatenating all vectors in the same spatial orientation as the original patches yielded a feature tensor, which we called a feature-quilt, that effectively compressed the initially massive image quilt into a compact representation useful for further downstream learning. Before concatenation, this feature-quilt was averaged to further compress the representation into a 128-dimensional feature vector for each patient. The tabular clinical data was concatenated with the output of the image pipeline. The concatenated vector was then fed to a CatBoost classifier^26^ and the model output a risk score for the task at hand.

### Assembling NRG/RTOG clinical trials data

With approval from NRG Oncology, a National Clinical Trials Network (NCTN) group funded by the National Cancer Institute (NCI), we assembled a unique dataset from five large multinational randomized phase III clinical trials of men with localized prostate cancer (NRG/RTOG-9202, 9408, 9413, 9910, and 0126)^20–24^. All patients received definitive external radiotherapy (RT), with or without pre-specified use of androgen-deprivation therapy (ADT). Combined RT with short-term ADT was of 4 month duration, with medium-term ADT of 36-week duration, and with long-term ADT of 28 month duration (Table 1). Of the 7,764 eligible patients randomized in these five trials, there were 5,654 with high quality digital histopathology image data. This represented 16.1 TB of histopathology image data from 16,204 histopathology slides of pretreatment and posttreatment prostate tissue.

**Table 1:** Clinicopathologic and trial characteristics.

	RTOG-9202	RTOG-9408	RTOG-9413	RTOG-9910	RTOG-0126	Combined
Number of Patients	1180	1719	695	976	1084	5654
*White*	1004	1312	481	769	937	4503
*Hispanic*	18	48	23	23	0	112
*African American*	147	334	173	166	112	932
*Asian*	4	12	1	11	12	40
*Other Race*	1	10	11	6	9	37
*Unknown Race*	6	3	6	1	14	30
Number of Pathology Slides	3188	5472	2104	3075	2365	16204
Number of Clinical Variables	53	69	71	60	62	−
Therapy Randomization	RTS vs. RTL	RT vs. RTS	RTS 2 × 2^a^	RTS vs. RTM	RT vs. RT +	−
Patient Risk Groups	Inter. | High	Low | Inter. | High	Inter. | High	Inter. | High	Inter.	Low | Inter. | High
Primary Endpoint	Disease-free Survival	Overall Survival	Progression-free Survival	Prostate Cancer-specific Mortality	Overall Survival	−
Median Follow-up for Censored Patients (Years)	17.4	15.1	13.7	9.3	13.2	11.4
No. Patients Died	944	1154	504	297	505	3404
Trial Accrual Dates	1992–1995	1994–2001	1995–1999	2000–2004	2002–2008	1992–2008

The column ‘combined’ shows the characteristics of the final dataset with all five trials used for training and validation. ^a^RTOG-9413 randomized patients in a 2 × 2 fashion testing the effect of timing of ADT (before and during RT vs. starting after RT) and field size (prostate only vs. full pelvic RT). New acronyms: radiotherapy plus short/medium/long-term hormone therapy (RTS/RTM/RTL).

### Identifying human-interpretable self-supervised learning image features

**Fig. 2:**
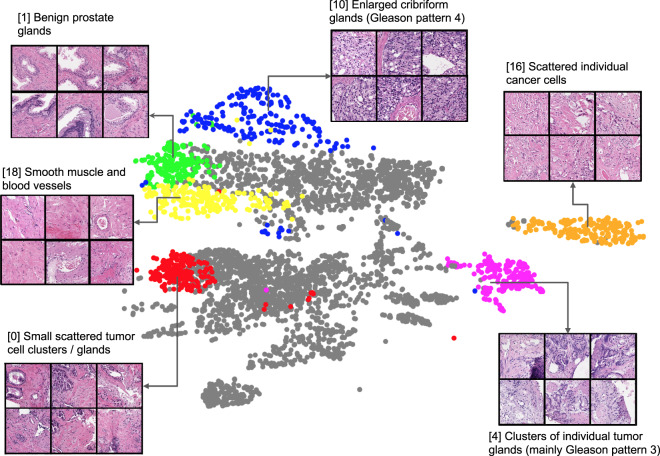
Pathologist interpretation of self-supervised model tissue clusters. The self-supervised model in the multimodal model was trained to identify whether or not augmented versions of small patches of tissue came from the same original patch, without ever seeing clinical data labels. After training, each image patch in the dataset of 10.05 M image patches was fed through this model to extract a 128-dimensional feature vector, and the UMAP algorithm^27^ was used to cluster and visualize the resultant vectors. A pathologist was then asked to interpret the 20 image patches closest to each of the 25 cluster centroids—the descriptions are shown next to the insets. For clarity, we only highlight 6 clusters (colored), and show the remaining clusters in gray. See Supplementary Fig. 2 for full pathologist annotation.

The internal data representations of the self-supervised learning model are shown in Fig. 2. We fed the entire dataset’s image patches through the self-supervised learning model and extracted model features—a 128-dimensional vector outputted by the model—for each patch. The Uniform Manifold Approximation and Projection algorithm (UMAP)^27^ was applied to these features, projecting them from 128 dimensions down to two, and each patch was plotted as an individual point. Neighboring data points represent image patches that the model considered similar. UMAP grouped the feature vectors into 25 clusters, some of which are shown in various colors, and a pathologist was asked to interpret the 20 nearest-neighbor image patches of the cluster centroids and try to identify trends observed for each cluster. Insets in Fig. 2 show example image patches (and pathologist descriptions) that are close in feature space to the cluster centroids, and the full interpretation for all 25 clusters is shown in Supplementary Fig. 2.

### Evaluating performance of the MMAI models on a validation set

The MMAI prognostic models developed using pathology images, NCCN variables (combined Gleason score, T-stage, baseline PSA), age, Gleason primary, and Gleason secondary, had superior discriminatory performance on the entire held-out test set across all clinical endpoints and timeframes when compared to the most commonly used NCCN risk-stratification tool.

**Fig. 3:**
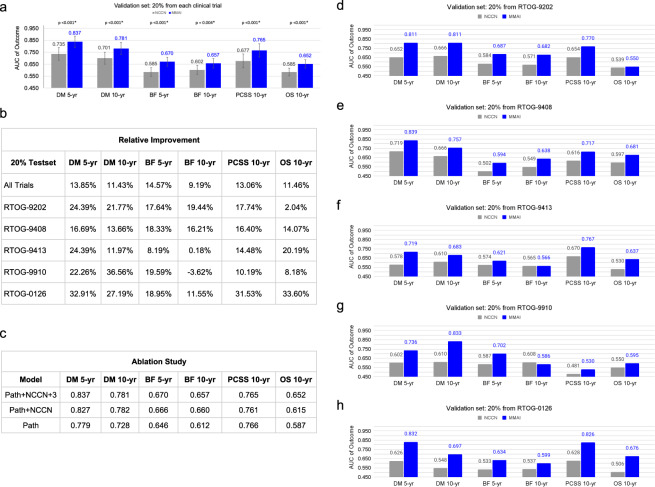
Comparison of the multimodal deep learning system to NCCN risk groups across trials and outcomes. **a** Performance results reporting on the area under the curve (AUC) of time-dependent receiver operator characteristics of the MMAI (blue bars) vs. NCCN (gray bars) models, include 95% confidence intervals and two-sided *p*-values. Comparisons were made across 5-year and 10-year time points on the following binary outcomes: distant metastasis (DM), biochemical failure (BF), prostate cancer-specific survival (PCSS), and overall survival (OS). **b** Summary table of the relative improvement of the MMAI model over the NCCN model across the various outcomes and broken down by performance on the data from each trial in the validation set. Relative improvement is given by (AUC_MMAI_ − AUC_NCCN_)/AUC_NCCN_. **c** Ablation study showing model performance when trained on a sequentially decreasing set of data inputs, including the pathology images only (path), pathology images + NCCN variables (path + NCCN), and pathology images + NCCN variables + age + Gleason primary + Gleason secondary (path + NCCN + 3). **d**–**h** Performance comparison on the individual clinical trial subsets of the validation set—together, these five comprise the entire validation set shown in (**a**).

The model performance results for the validation sets are shown in Fig. 3. In Figs. 3a and d–h, the blue bars represent the performance of the MMAI models, each trained on a specific endpoint timeframe, and the gray bars represent the performance of the corresponding NCCN model. Figure 3b shows the relative improvement of the MMAI over NCCN across the outcomes, and across the subsets of the validation set that come from the five individual trials. As can be seen, our model consistently outperforms the NCCN model across all tested outcomes, with a substantial relative improvement in AUC varying from 9.2% to 14.6%. Further, the trial subsets unanimously see a relative improvement over NCCN except for prediction of 10 year biochemical failure in RTOG-9910. This trial had one of the lower event rates and shortest follow-up times compared to the remaining trials, and all patients received hormone therapy. With a short follow-up time, patients were less likely to recover their testosterone and prostate-specific antigen levels to be able to experience biochemical failure.

To evaluate the incremental benefit of various data components, we ran an ablation study in which we sequentially removed data features to understand their effect on the overall performance of the system^28^. We trained additional MMAI models using the following data setups: pathology images only, pathology images + the NCCN variables (combined Gleason score, T-stage, baseline PSA), and pathology images + NCCN variables + three additional variables (age, Gleason primary, Gleason secondary). Each additional data component improved performance, with the full setup (pathology images and six clinical variables) yielding the best results (Fig. 3c). The MMAI prognostic model had superior discrimination compared to the NCCN model for all outcomes, including 5-year distant metastasis (AUC of 0.84 vs. 0.74, *p*-value < 0.001), 5-year biochemical failure (AUC of 0.67 vs. 0.59, *p*-value < 0.001), 10-year prostate cancer-specific survival (AUC of 0.77 vs. 0.68, *p*-value < 0.001), and 10-year overall survival (AUC of 0.65 vs. 0.59, *p*-value < 0.001).

In a subsequent analysis designed to assess the model in the intended use setting, we repeated the evaluation of model performance in the validation set after removing any patients with posttreatment prostate tissue (*n* = 931; 71% of removed patients were from NRG/RTOG 9408). Performance evaluation results are summarized in Table 2. The MMAI prognostic model had superior discrimination compared to the NCCN model for all outcomes, including 5-year distant metastasis (AUC of 0.83 vs 0.72, *p*-value < 0.001), 5-year biochemical failure (AUC of 0.69 vs 0.61, *p*-value < 0.001), 10-year prostate cancer-specific survival (AUC of 0.77 vs 0.67, *p*-value < 0.001), and 10-year overall survival (AUC of 0.65 vs 0.57, *p*-value < 0.001).

**Table 2:** Validation results for the subset of patients from the 20% validation set that includes patients with pretreatment slides only (*n* = 931).

Clinical outcome	NCCN AUC estimates (95% CI)	MMAI AUC (95% CI)	Differential AUC estimate (MMAI - NCCN)	Comparative test *p*-value
Distant Metastasis (5-year)	0.72 (0.67–0.78)	0.83 (0.78–0.88)	0.11	<0.001
Distant Metastasis (10-year)	0.69 (0.64–0.74)	0.78 (0.73–0.84)	0.09	<0.001
Biochemical Failure (5-year)	0.61 (0.57–0.64)	0.69 (0.65–0.73)	0.08	<0.001
Biochemical Failure (10-year)	0.62 (0.58–0.66)	0.68 (0.63–0.72)	0.06	0.004
Prostate Cancer-Specific Survival (10-year)	0.67 (0.61–0.73)	0.77 (0.70–0.83)	0.10	<0.001
Overall Survival (10-year)	0.57 (0.54–0.61)	0.65 (0.61–0.69)	0.08	<0.001

*AUC* Area under the curve, *CI* confidence interval*, MMAI* multimodal artificial intelligence, *NCCN* National Comprehensive Cancer Network.

## Discussion

Prior work prognosticating outcomes from histopathology slides typically leverages extensive region-level pathologist annotations on slides (e.g., to train AI models to predict Gleason grading^19,29,30^, or to prognosticate short-term outcomes^18^). In contrast, our technique learns from patient-level clinical data and unannotated histopathology slides and can prognosticate long-term outcomes. Moreover, the self-supervised learning of the image model allows it to learn from new image data without the need for additional annotations. When deploying machine learning (ML) models in a new domain (e.g., a new scanner or a new clinic) including data from that domain during training can help improve generalization and performance. This system also lowers the barrier for physicians to begin using this tool and for clinics to easily continue sharing histopathology image data to obtain prognostic information and aid their treatment decisions. This lower barrier for usage is a valuable advantage when deploying the system in new locations and attempting to adapt to their inherent biases—a challenge previously observed in medical AI deployment^31^. In addition, this tool focuses on supporting the oncologist in making treatment decisions and provides complementary information to the diagnostic and histopathology information identified by a pathologist.

Self-supervised learning^25^ is a method recently popularized in the ML community for learning from datasets without annotations. Typical ML setups leverage supervised learning, in which datasets are composed of data points (e.g., images) and data labels (e.g., object classes). In contrast, during self-supervised learning, *synthetic* data labels are *extracted* from the original data, and used to train generic feature representations which can be used for downstream tasks. Here we find that momentum contrast^32^—a technique that takes the set of image patches, generates augmented copies of each patch, then trains a model to predict whether any two augmented copies come from the same original patch—is effective at learning features from digital pathology slides. The structural setup is shown in Fig. 1b, with further details in the Methods. One challenge with real-world medical datasets is the sheer volume of image data available and potential class imbalance (tissue vs. no tissue) that is a result of how histopathology slides are created. To overcome this and guide the self-supervised learning process towards patch regions that are likely to be more clinically useful, we only use images from patients with a Gleason primary ≥4. Future work could investigate further whether other training techniques such as transfer learning are effective, and whether training or fine-tuning models end-to-end using patient-level information improves final performance.

When new models are introduced, an understanding of how to use them in routine clinical care is critical to the adoption of such tools. In this study, we focus on the unique AI architecture used to develop the prognostic models trained on data from thousands of patients with accurate, long-term follow-up data and clinically relevant outcomes. The MMAI model consistently outperforms the NCCN model across all tested outcomes in the intended use validation set (i.e., pretreatment tissue only). However, further work will be required to evaluate the clinical utility of this model, including identifying actionable information from defined patient risk groups and calibration with contemporary data, and omitting posttreatment tissue. Importantly, interpretability of the features used by the model to predict prognosis should be investigated, and will be the subject of future work.

By creating a deep learning architecture that simultaneously ingests multiple data types, including histopathology image data of variable sizes as well as clinical data, we built a deep learning system capable of inferring long-term patient outcomes that substantially outperforms established clinical models. This study leverages robust and large-scale clinical data from five prospective, randomized, multinational phase III trials with up to 20 years of patient follow-up for 5,654 patients across a varied population, enrolled at hundreds of different diverse medical centers. Validation of these prognostic classifiers on a large amount of clinical trials data—in the intended use population—uniquely positions these tools as aids to therapeutic decision-making. Barriers to the adoption of urine-, blood-, and tissue-based molecular assays include their invariably high costs, the collection and consumption of biospecimen samples, and long turnaround times. In contrast, AI tools lack these limitations, substantially lowering their barrier to large-scale adoption. Moreover, the growing adoption of digital histopathology will support the global distribution of AI-based prognostic and predictive testing. This will enable broad access to therapy personalization and enable AI algorithms to continue improving by learning from diverse multinational data.

## Methods

### Dataset preparation

In collaboration with NRG Oncology, we obtained access to full patient-level baseline clinical data, digitized histopathology slides of pretreatment and posttreatment prostate tissue, and longitudinal outcomes from five landmark, large-scale, prospective, randomized, multinational clinical trials containing 5654 patients, 16,204 histopathology slides, and >10 years of median follow-up: NRG/RTOG-9202, 9408, 9413, 9910, and 0126 (Table 1). Patients in these trials were randomized across various combinations of external radiotherapy (RT) with or without different durations of androgen-deprivation therapy (ADT). The slides were digitized over a period of 1 year by NRG Oncology using a Leica Biosystems Aperio AT2 digital pathology scanner at a resolution of 20x. The histopathology images were manually reviewed for quality and clarity. Six baseline clinical variables that were collected across all trials (combined Gleason score, Gleason primary, Gleason secondary, T-stage, baseline PSA, age), along with the digital histopathology images, were used for model training and validation. The patients from five trials were split into training (80%) and validation (20%) datasets, and there was no patient overlap among splits. To ensure that the test set captured a clinically relevant and representative subset of patients, the final test set was selected such that the NCCN risk group’s 5-year distant metastasis AUC performance was between 0.7 and 0.75, as observed in the literature^33,34^. Institutional Review Board approval was obtained from NRG Oncology (IRB00000781) and informed consent was waived because this study was performed with anonymized data.

### Image pipeline

All tissue from digitized slides were segmented into a single image quilt of size 200 by 200 patches for each patient prior to model training. A simple grid was then laid over the image quilt to obtain contiguous and adjacent patches of size 256 × 256 pixels. We used a ResNet-50 model^35^, together with the MoCo-v2 training protocol^36^ (parameters: learning rate = 0.03 with a cosine learning rate schedule for 200 epochs, moco-t = 0.2, multilayer perceptron head, batch size of 256, the default MoCo-v2 parameters for augmentation), to train the self-supervised learning model used in the system architecture of Fig. 1b. For the validation results shown in Fig. 3a, we used images of patients with a Gleason primary ≥4 to pre-train a corresponding self-supervised learning model to effectively learn relevant histomorphologic features. Once self-supervised pre-training was complete, we fed in all patches with usable tissue (See tissue segmentation section) to the self-supervised pretrained ResNet-50 model to generate an image feature vector for each patch. These image feature vectors were averaged to produce a 128-dimensional image feature for each patient.

### Fusion pipeline

To leverage information from both modalities (image and clinical features), we used a joint fusion approach. The tabular clinical data were all considered as numerical variables, and a CatBoost^26^ model that took in a concatenation of numerical clinical variables and image features as input was used for model prediction and to output a risk score (parameters: learning rate 0.003, depth 5, L2 leaf regularization 10, 2000 iterations).

### Tissue segmentation

After the slides were cut into 256 × 256 pixel patches, we developed an artifact classifier by training a ResNet-18 to classify whether a patch showed usable tissue, or whether it showed whitespace or artifacts. The artifact classifier was trained for 25 epochs, optimized using stochastic gradient descent with a learning rate of 0.001. The learning rate was reduced by 10% every 7 epochs. We manually annotated 3661 patches (tissue vs. not tissue) and trained this classifier on 3366 of them, achieving a validation accuracy of 97.6% on the remaining patches. This artifact classifier was then used to segment tissue sections and filter out low-quality images during image feature generation.

### Model performance metrics (AUC)

For each model and each outcome, we estimated the time-dependent receiver operating characteristic curve, accounting for competing events and censoring, using the R-package timeROC^37^. The area under this curve defines the model’s performance. Each time-dependent curve was constructed by evaluating the sensitivities and specificities based on the disease statuses fixed at time *t* and the model predictions determined by sweeping through a threshold *c*. Methods detailed in Blanche et al. were used to compute pointwise 95% confidence intervals (1.96 × standard error) for AUCs and two-sided *p*-values for comparing AUCs of two models (e.g., MMAI vs. NCCN)^37^.

### NCCN risk groups

Three variables—clinical T-stage, Gleason score, and baseline PSA—were used to group patients into low-, intermediate-, and high-risk groups. The risk groups were defined as follows: low risk (cT1–cT2a, Gleason score ≤6, and PSA <10 ng/mL), intermediate risk (cT2b–cT2c, Gleason score 7, and/or PSA 10–20 ng/mL), and high risk (≥cT3a or Gleason score 8–10 or PSA >20 ng/mL)^9^.

### Reporting summary

Further information on research design is available in the Nature Research Reporting Summary linked to this article.

## Supplementary information

Supplementary Figures

Reporting Summary Checklist

## Data Availability

The data published in this article will be publicly available six months from publication, through requests made to NRG Oncology at APC@nrgoncology.org.

## Code availability

The multimodal AI architecture was developed using PyTorch Python library (https://pytorch.org/). In addition, scikit-learn, NumPy, statsmodels, pandas, Matplotlib, and MoCo-v2 have been used for computation and plotting (available under: https://scikit-learn.org/stable/, https://numpy.org/, https://www.statsmodels.org/, https://pandas.pydata.org/, https://matplotlib.org/, and https://github.com/facebookresearch/moco). The trained model used in this study has not yet undergone regulatory review and cannot be made available at this time. Interested researchers can contact A.E. (aesteva@artera.ai) for questions on its status and access.
