# Author Correction: Prostate cancer therapy personalization via multi-modal deep learning on randomized phase III clinical trials

**DOI:** 10.1038/s41746-023-00769-z

**Published:** 2023-02-22

**Authors:** Andre Esteva, Jean Feng, Douwe van der Wal, Shih-Cheng Huang, Jeffry P. Simko, Sandy DeVries, Emmalyn Chen, Edward M. Schaeffer, Todd M. Morgan, Yilun Sun, Amirata Ghorbani, Nikhil Naik, Dhruv Nathawani, Richard Socher, Jeff M. Michalski, Mack Roach, Thomas M. Pisansky, Jedidiah M. Monson, Farah Naz, James Wallace, Michelle J. Ferguson, Jean-Paul Bahary, James Zou, Matthew Lungren, Serena Yeung, Ashley E. Ross, NRG Prostate Cancer AI Consortium, Michael Kucharczyk, Luis Souhami, Leslie Ballas, Christopher A. Peters, Sandy Liu, Alexander G. Balogh, Pamela D. Randolph-Jackson, David L. Schwartz, Michael R. Girvigian, Naoyuki G. Saito, Adam Raben, Rachel A. Rabinovitch, Khalil Katato, Howard M. Sandler, Phuoc T. Tran, Daniel E. Spratt, Stephanie Pugh, Felix Y. Feng, Osama Mohamad

**Affiliations:** 1 Artera, Inc, Mountain View, CA USA; 2 Department of Epidemiology and Biostatistics, University of California San Francisco, San Francisco, CA USA; 3 Department of Biomedical Data Science, Stanford University, Stanford, CA USA; 4 Department of Radiation Oncology, University of California San Francisco, San Francisco, CA USA; 5 NRG Oncology Biospecimen Bank, San Francisco, CA USA; 6 Department of Urology, Northwestern University, Evanston, IL USA; 7 Division of Urologic Oncology, University of Michigan Comprehensive Cancer Center, Ann Arbor, MI USA; 8 Department of Population and Quantitative Health Sciences, Case Western Reserve University, Cleveland, OH USA; 9 Salesforce Research, San Francisco, CA USA; 10 Department of Radiation Oncology, Washington University School of Medicine, Saint Louis, MO USA; 11 Department of Radiation Oncology, Mayo Clinic, Rochester, MN USA; 12 Department of Radiation Oncology, cCare, Fresno, CA USA; 13 Department of Radiation Oncology, Horizon Health Network-Saint John Regional Hospital, Saint John, JB E2L 4L2 CA Canada; 14 Department of Hematology and Oncology, Ingalls Memorial Hospital, Harvey, IL USA; 15 Department of Radiation Oncology, Allan Blair Cancer Centre, Regina, SK S4T 7T1 CA Canada; 16 Department of Radiation Oncology, CHUM - Centre Hospitalier de l’Universite de Montreal, Montreal, QC H2X 3E4 CA Canada; 17 Department of Radiation Oncology, Cedars-Sinai Medical Center, Los Angeles, CA USA; 18 Department of Radiation Oncology, University of Maryland, Baltimore, MD USA; 19 Department of Radiation Oncology, University Hospitals Seidman Cancer Center, Case Western Reserve University, Cleveland, OH USA; 20 NRG Oncology Statistics and Data Management Center, Philadelphia, PA USA; 21 Department of Radiation Oncology, Accrual-Nova Scotia Cancer Centre, Halifax, Nova Scotia B3H 1V7 Canada; 22 Department of Oncology, The Research Institute of the McGill University Health Centre (MUHC), Montreal, QC H4A 3J1 CA Canada; 23 Department of Radiation Oncology, University of Southern California, Los Angeles, CA USA; 24 Department of Radiation Oncology, Northeast Radiation Oncology Centers (NROC), Dunmore, PA USA; 25 Department of Medical Oncology, UCLA Medical Center, Los Angeles, CA USA; 26 Department of Radiation Oncology, Tom Baker Cancer Centre, Calgary, AB T2N 4N2 CA Canada; 27 Department of Radiation Oncology, Washington Cancer Institute, Washington, DC USA; 28 Department of Radiation Oncology, Veteran Affairs New York Harbor Healthcare System-Brooklyn Campus, Brooklyn, NY USA; 29 Department of Radiation Oncology, Kaiser Permanente Los Angeles Medical Center, Los Angeles, CA USA; 30 Department of Radiation Oncology, Indiana University School of Medicine, Indianapolis, IN USA; 31 Department of Radiation Oncology, Christiana Care Health System-Christiana Hospital, Newark, DE USA; 32 Department of Radiation Oncology, University of Colorado Hospital, Aurora, CO USA; 33 Department of Medical Oncology, Hurley Medical Center, Flint, MI USA

**Keywords:** Prognostic markers, Prostate cancer, Machine learning

Correction to: *npj Digital Medicine* 10.1038/s41746-022-00613-w, published online 08 June 2022

The original version of the published Article contained an error in the description of the prostate tissue samples in the Methods and Results sections, which stated that “pretreatment biopsy samples” and “pretreatment prostate biopsies” were used in this study. Both instances have been updated to “pretreatment and posttreatment prostate tissue”, and these changes are reflected in the HTML and PDF versions of the Article. Furthermore, we have added descriptions of the use of posttreatment prostate tissue during model development throughout the Article.

In the interest of transparency and clinical relevance, we repeated the evaluation of model performance in the validation set after removing any cases with post-treatment prostate tissue. This results in a slightly smaller cohort (*n* = 931) for validation, compared to the 20% validation set used in the original paper, and still demonstrates similar performance to the MMAI algorithms using only pretreatment tissue for each clinical endpoint. Table 2 has been added to the HTML and PDF versions of the Article.

**Table 2:** Validation results for the subset of patients from the 20% validation set reported in the Article that includes patients with pretreatment slides only (*n* = 931).

Clinical outcome	NCCN AUC estimates (95% CI)	MMAI AUC (95% CI)	Differential AUC estimate (MMAI - NCCN)	Comparative test *p* value
Distant metastasis (5-year)	0.72 (0.67–0.78)	0.83 (0.78–0.88)	0.11	<0.001
Distant metastasis (10-year)	0.69 (0.64–0.74)	0.78 (0.73–0.84)	0.09	<0.001
Biochemical failure (5-year)	0.61 (0.57–0.64)	0.69 (0.65–0.73)	0.08	<0.001
Biochemical failure (10-year)	0.62 (0.58–0.66)	0.68 (0.63–0.72)	0.06	0.004
Prostate cancer-specific survival (10-year)	0.67 (0.61–0.73)	0.77 (0.70–0.83)	0.10	<0.001
Overall survival (10-year)	0.57 (0.54–0.61)	0.65 (0.61–0.69)	0.08	<0.001

The subcohort represented in this table is smaller than reported in the Article (*n* = 1109).

*AUC* area under the curve, *CI* confidence interval, *MMAI* multimodal artificial intelligence, *NCCN* National Comprehensive Cancer Network.

## Data Availability

The data availability statement has also been updated in the PDF and HTML versions of the Article to read, “The data published in this article will be publicly available six months from publication, through requests made to NRG Oncology at APC@nrgoncology.org”.

