# Supplementary material for: Prostate cancer therapy personalization via multi-modal deep learning on randomized phase III clinical trials
Source: NPJ Digit Med. 2022 Jun 8;5:71. doi: 10.1038/s41746-022-00613-w (PMC9177850; doi:10.1038/s41746-022-00613-w)
Supplement: Supplementary file 1 — Supplementary Figures [file 41746_2022_613_MOESM1_ESM.pdf]

Supplementary Figures

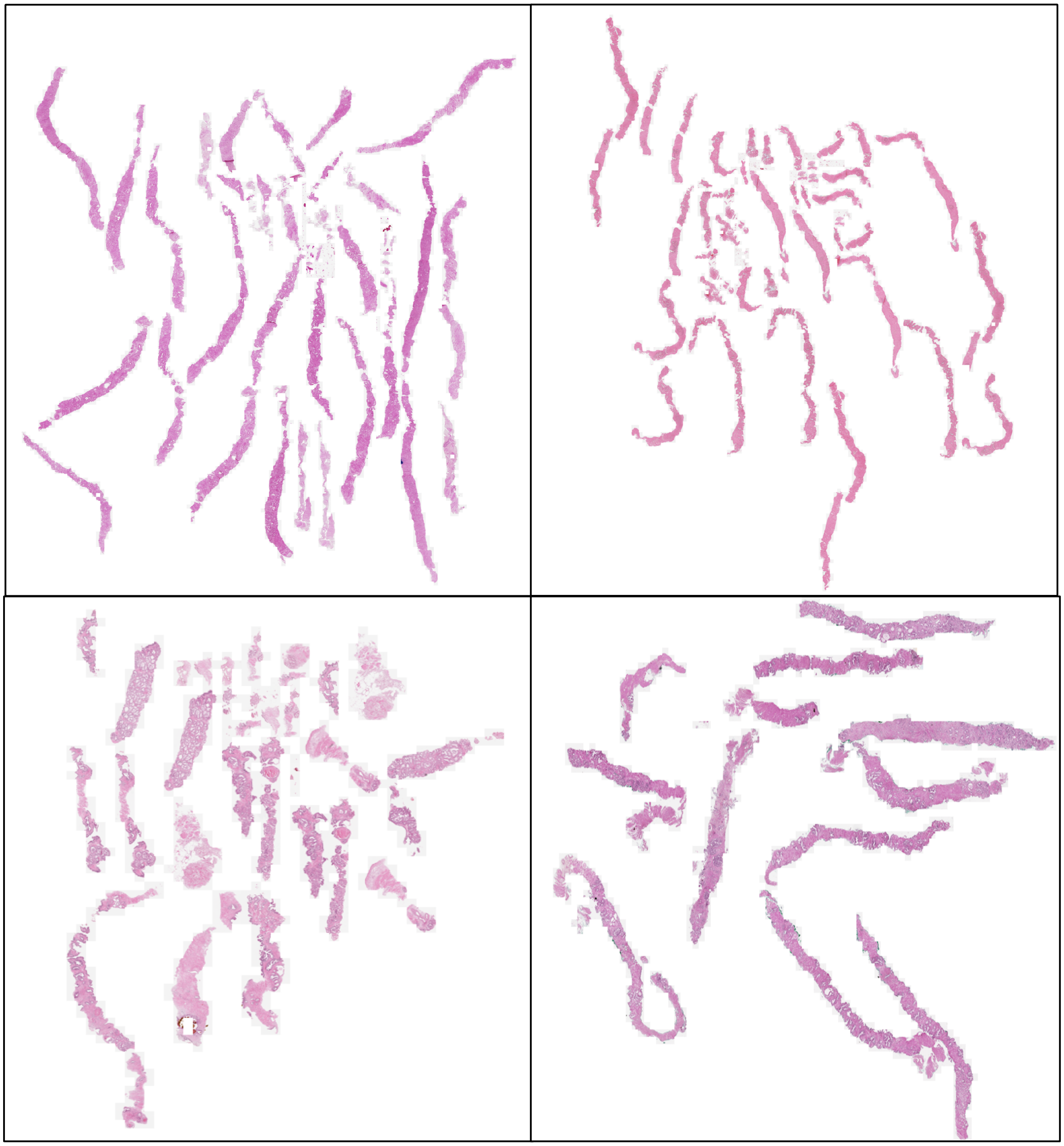

Supplementary Figure 1. Image quilts for four example patients. The dataset used in this study included patients with a variable number of histopathology slides. To standardize the image inputs to the model, the tissue from each slide was segmented, and all tissues were pasted into a single square image of 51200 x 51200 pixels and divided into 200 by 200 patches, representing all the histopathology data of a single patient.

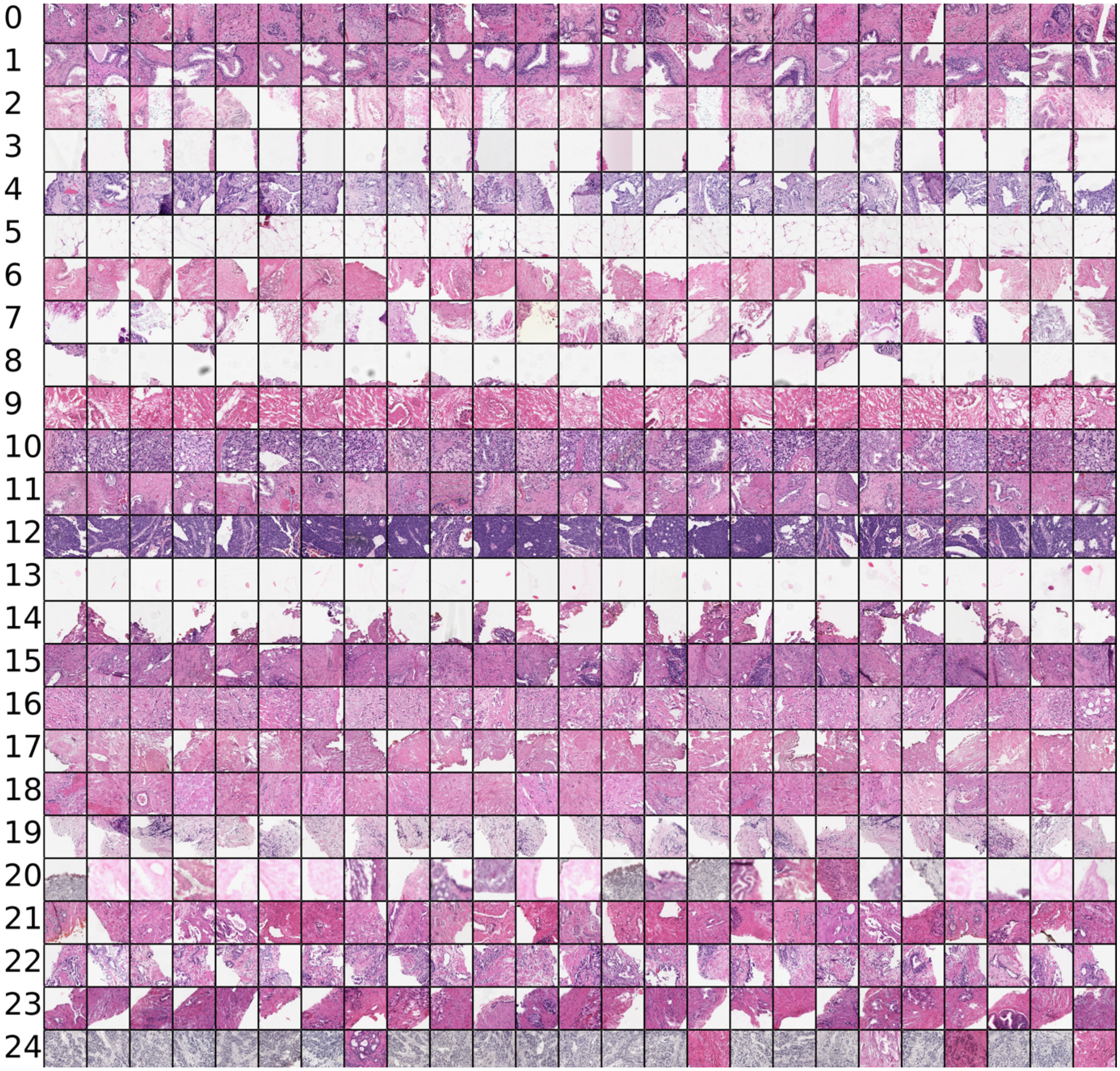

0	Small scattered tumor cell clusters / glands (various Gleasons)
1	Benign prostate glands
2	Scanning artifacts
3	Mostly whitespace
4	Clusters of individual tumor glands (mainly Gleason pattern 3)
5	Fat tissue
6	Prostate smooth muscle, occasional tumor glands and occasional benign glands
7	Fibro-muscular stroma
8	Small edges of tissue
9	Prostate smooth muscle, and occasional benign glands
10	Enlarged cribriform glands (Gleason pattern 4)

11	Scattered individual glands most likely benign, with prostate smooth muscle tissue
12	Large sheets of cancer cells (Gleason patterns 4 or 5)
13	Dead cells and artifacts
14	Cauterized edge of stroma
15	Prostate smooth muscle
16	Scattered individual cancer cells
17	Prostate smooth muscle
18	Smooth muscle and blood vessels
19	Loose stroma with scattered crust or small cells
20	Out of focus cluster
21	Prostate smooth muscle with scattered individual prostate glands that are mostly malignant
22	Glands of varying sizes, potentially benign or malignant
23	Prostate smooth muscle, with small, crushed glands, some are cancerous, some benign
24	Scattered cells and glands with atrophic features (Gleason patterns 4 and 3)

Supplementary Figure 2. Pathologist-interpreted patch clusters. Using UMAP, 25 clusters were generated from the self-supervised model features of all the histopathology patches from RTOG 9202. Each row in the image corresponds to the 25 nearest-neighbor image patches of the cluster centroid. These were inspected by a pathologist to determine the human-interpretable descriptions of the clusters listed in the table.
