# Supplementary material for: Prostate cancer therapy personalization via multi-modal deep learning on randomized phase III clinical trials
Source: NPJ Digit Med. 2022 Jun 8;5:71. doi: 10.1038/s41746-022-00613-w (PMC9177850; doi:10.1038/s41746-022-00613-w)
Supplement: Supplementary file 2 — Reporting Summary Checklist [file 41746_2022_613_MOESM2_ESM.pdf]

nature portfolio

Corresponding author(s): Andre Esteva (NPJDIGITALMED-03305)

Last updated by author(s): Apr 17, 2022

Reporting Summary

Nature Portfolio wishes to improve the reproducibility of the work that we publish. This form provides structure for consistency and transparency in reporting. For further information on Nature Portfolio policies, see our Editorial Policies and the Editorial Policy Checklist.

Statistics

For all statistical analyses, confirm that the following items are present in the figure legend, table legend, main text, or Methods section.

n/a	Confirmed	
☐	☒	The exact sample size (*n*) for each experimental group/condition, given as a discrete number and unit of measurement
☐	☒	A statement on whether measurements were taken from distinct samples or whether the same sample was measured repeatedly
☐	☒	The statistical test(s) used AND whether they are one- or two-sided<br>*Only common tests should be described solely by name; describe more complex techniques in the Methods section.*
☐	☒	A description of all covariates tested
☐	☒	A description of any assumptions or corrections, such as tests of normality and adjustment for multiple comparisons
☐	☒	A full description of the statistical parameters including central tendency (e.g. means) or other basic estimates (e.g. regression coefficient) AND variation (e.g. standard deviation) or associated estimates of uncertainty (e.g. confidence intervals)
☐	☒	For null hypothesis testing, the test statistic (e.g. *F*, *t*, *r*) with confidence intervals, effect sizes, degrees of freedom and *P* value noted<br>*Give P values as exact values whenever suitable.*
☒	☐	For Bayesian analysis, information on the choice of priors and Markov chain Monte Carlo settings
☒	☐	For hierarchical and complex designs, identification of the appropriate level for tests and full reporting of outcomes
☒	☐	Estimates of effect sizes (e.g. Cohen's *d*, Pearson's *r*), indicating how they were calculated

Our web collection on statistics for biologists contains articles on many of the points above.

Software and code

Policy information about availability of computer code

Data collection	Not applicable since this is a retrospective research on completed studies. Data were provided by NRG Oncology.
Data analysis	The multi-modal AI architecture was developed using PyTorch Python library (https://pytorch.org/). In addition, scikit-learn, NumPy, statsmodels, pandas, Matplotlib, and MoCo-v2 have been used for computation and plotting (available under: https://scikit-learn.org/stable/, https://numpy.org/, https://www.statsmodels.org/, https://pandas.pydata.org/, https://matplotlib.org/, and https://github.com/facebookresearch/moco). Time-dependent area under the ROC curve analyses were performed using an R package, timeROC.

For manuscripts utilizing custom algorithms or software that are central to the research but not yet described in published literature, software must be made available to editors and reviewers. We strongly encourage code deposition in a community repository (e.g. GitHub). See the Nature Portfolio guidelines for submitting code & software for further information.

Data

Policy information about availability of data

All manuscripts must include a data availability statement. This statement should provide the following information, where applicable:

- Accession codes, unique identifiers, or web links for publicly available datasets
- A description of any restrictions on data availability
- For clinical datasets or third party data, please ensure that the statement adheres to our policy

The data that support the findings of this study included pathology slides, clinicopathologic variables, and outcomes information from NRG Oncology. Data may be made available for noncommercial academic use from the authors with permission from NRG Oncology. For access to the clinicopathology variables and outcomes information, please contact AP@nrgoncology.org. For the digitized pathology slides, please contact A.E. (aesteva@artera.ai).

Field-specific reporting

Please select the one below that is the best fit for your research. If you are not sure, read the appropriate sections before making your selection.

☒ Life sciences ☐ Behavioural & social sciences ☐ Ecological, evolutionary & environmental sciences

For a reference copy of the document with all sections, see nature.com/documents/nr-reporting-summary-flat.pdf

Life sciences study design

All studies must disclose on these points even when the disclosure is negative.

Sample size	This is a retrospective research using completed studies. We included all the patients with image data available. No sample size justification was performed because no archived tissues were consumed and the analyses did not pose additional risks to patients.
Data exclusions	We excluded patients that were not able to contribute digital histopathologic images.
Replication	This is a retrospective researching using AI technology. The AI architecture enables reproducibility of the models and findings.
Randomization	This is not relevant for this retrospective research.
Blinding	This is not relevant for this retrospective research.

Reporting for specific materials, systems and methods

We require information from authors about some types of materials, experimental systems and methods used in many studies. Here, indicate whether each material, system or method listed is relevant to your study. If you are not sure if a list item applies to your research, read the appropriate section before selecting a response.

Materials & experimental systems

n/a	Involved in the study	
☒	☐	Antibodies
☒	☐	Eukaryotic cell lines
☒	☐	Palaeontology and archaeology
☒	☐	Animals and other organisms
☐	☒	Human research participants
☐	☒	Clinical data
☒	☐	Dual use research of concern

Methods

n/a	Involved in the study	
☒	☐	ChIP-seq
☒	☐	Flow cytometry
☒	☐	MRI-based neuroimaging

Human research participants

Policy information about studies involving human research participants

Population characteristics	Five large multinational randomized phase III clinical trials of men with intermediate-high risk localized prostate cancer (NRG/RTOG 9202, 9408, 9413, 9910, and 0126). All patients received definitive external radiotherapy (RT), with or without pre-specified use of androgen-deprivation therapy (ADT).
Recruitment	Please refer to the original primary result publications for each study (NRG/RTOG 9202, 9408, 9413, 9910, and 0126).
Ethics oversight	Late Phase IRB00000781 Phase II, II/III, and III

Note that full information on the approval of the study protocol must also be provided in the manuscript.

Clinical data

Policy information about clinical studies

All manuscripts should comply with the ICMJE guidelines for publication of clinical research and a completed CONSORT checklist must be included with all submissions.

Clinical trial registration	RTOG 9408 - NCT00002597, RTOG 9413 - NCT00769548, RTOG 0126 - NCT00033631, RTOG 9212 - NCT00767286, RTOG 9910 - NCT00005044
Study protocol	All study protocols can be found through CTSU (https://www.ctsu.org).

Data collection	Data were provided by NRG/RTOG. Please refer to the original primary result publications for each study (NRG/RTOG 9202, 9408, 9413, 9910, and 0126).
Outcomes	Outcome data were provided by NRG/RTOG.
